# A Multimodal Evaluation of Podcast Learning, Retention, and Electroencephalographically Measured Attention in Medical Trainees

**DOI:** 10.7759/cureus.31289

**Published:** 2022-11-09

**Authors:** Jed Wolpaw, Sahin Ozsoy, Sean Berenholtz, Scott Wright, Kelly Bowen, Shravya Gogula, Sehyun Lee, Serkan Toy

**Affiliations:** 1 Anesthesiology and Critical Care Medicine, Johns Hopkins University School of Medicine, Baltimore, USA; 2 Bioengineering, BioSoftPro, Baltimore, USA; 3 Internal Medicine, Johns Hopkins Bayview Medical Center, Baltimore, USA; 4 Medicine, Cleveland Clinic Lerner College of Medicine, Case Western Reserve University, Cleveland, USA; 5 Medicine, Georgetown University School of Medicine, Washington DC, USA; 6 Basic Science Education, Virginia Tech Carilion School of Medicine, Roanoke, USA

**Keywords:** attention, assessment, asynchronous education, podcasts, medical education

## Abstract

Introduction: Podcasts have become popular among medical trainees. However, it is unclear how well learners retain information from podcasts compared to traditional educational modalities, and whether multitasking affects the learner’s ability to pay attention and learn. This study attempted to examine the effectiveness of podcast learning by using electroencephalography (EEG) to measure learner attention, in addition to test performance, task load, and preferences.

Methods: The study used a repeated measures design with three conditions: podcast listening on a treadmill, podcast listening seated, and textbook reading seated. Participants were anesthesiology residents and medical students at a large United States academic medical center. Three topics were chosen: allergic response, liver physiology, and statistics. Each participant studied all three topics that were randomly assigned to one of three learning conditions - in random order. Participants completed a knowledge test at baseline, after each condition, and at four-week follow-up, and reported preferred learning modality and task load under each modality. Activation levels in alerting, orienting, and executive attentional networks were examined using EEG.

Results: Sixty-one participants (11 anesthesiology residents and 50 medical students) were included in the study. Of the 61, six were excluded from the EEG analyses due to corrupted recordings. EEG results showed that mean attention network activation scores did not differ between the study conditions. Trainees preferred podcast learning over reading for all three topics. When compared to textbook reading, podcast learning (seated or on a treadmill) produced significantly better learning gain, and equivalent retention for two of the three topics.

Conclusions: Our study is the first to use neurocognitive data, self-reported satisfaction, and knowledge test performance to demonstrate that podcasts are at least equivalent to textbooks for maintaining attention, immediate learning, and retention - even while exercising.

## Introduction

Technological advances continue to offer opportunities for educators to expand their pedagogy beyond the confines of the traditional classroom. Podcasts are one example of such technology and have become increasingly popular [1]. The popularity of podcast education may be due to, in part, the fact that resident physicians are struggling to find ways to balance learning, service, and their personal lives. Podcasts allow them to learn while exercising or commuting, without adding any time to their already busy schedule.

One of the criticisms of podcasts is that they are often used while multitasking, raising concerns that learners may not pay full attention to the recording such that learning may suffer [2]. Some evidence has suggested that multitasking could increase cognitive load and hinder learning [3]. However, one study showed that learners retain more information when they are doing something mindless that does not require higher-order thinking skills, like doodling, than when they are not [4]. Multiple studies have also shown that aerobic exercise, especially immediately after learning, increases retention [5].

Meanwhile, educational research is playing catch-up to provide sound evidence for learning technologies in this fast-moving digital era. Within the literature, self-report surveys have been the primary source of evidence for measuring the popularity and effectiveness of podcast learning. Despite advances in neuroimaging technology over the last two decades, use of neurocognitive assessment metrics, such as electroencephalography (EEG), in medical education research has been limited. To date, a small but growing body of research illustrates that neurocognitive evidence is effective in studying psychomotor skill development, clinical reasoning, visual expertise development, and neural correlates of resident well-being and burnout [6]. EEG offers a viable continuous measure of neurocognitive activities with high temporal resolution on the order of milliseconds when examining task-related online cognitive engagement [6]. Fan demonstrated that the three main attentional networks in the brain (alerting, orienting, and executive attention) are linked to separate, identifiable brain regions [7,8]. To our knowledge, EEG measurement of attention in these three networks has not been used to evaluate podcasts as a learning modality.

We hypothesized that podcasts would be superior to textbooks in terms of (1) learner preference, (2) learner performance on testing, and (3) EEG measurement of attention. We also hypothesized that listening to a podcast on a treadmill, rather than while sitting, would result in better learning, better retention, and increased attention as indicated by EEG measurement of the three attentional networks.

## Materials and methods

Study design

This study used a repeated measures design with the following three learning conditions: (1) podcast listening while on a treadmill, (2) podcast listening while seated, and (3) textbook chapter reading while seated.

Educational materials

Three anesthesiology-related, complicated topics were selected - allergic response, liver physiology, and statistics. These topics were chosen after consulting a panel of experts in anesthesiology education. One of the study team members (JW), who has five years of experience creating podcasts, developed a podcast for each of the topics. These podcasts were approximately 45 minutes in length. For each of the topics, a standard textbook chapter was also selected from Clinical Anesthesia, eighth edition [9].

Participants and procedures

This study was conducted at a large urban tertiary care academic medical center in the Mid-Atlantic region of the United States. Our anesthesiology residency program has 80 residents across three years and the medical school has approximately 450 students across all four years. Recruitment was open to all anesthesiology residents and medical students. The study team sent out recruitment emails to all anesthesiology residents and medical students. Power analysis indicated that 57 participants would yield 90% power to detect a 0.20 effect size for this repeated measures design with three learning conditions, including baseline, posttest, and four-week follow-up knowledge tests with an alpha level=0.05. We closed enrollment at 65 participants.

A reminder email was sent to participants the Friday before the week of the study with an explanation of the tasks and necessary equipment to bring, including a personal electronic device for completing online questionnaires and knowledge tests, as well as headphones for listening to podcasts. Participants completed a total of three learning sessions, each covering one of the three topics. Topics were randomly assigned to one of the three learning conditions, and participants completed them in a random order to counteract the order effect.

After verbal consent was obtained, participants completed online baseline tests to assess their pre-session knowledge of the three learning topics, as well as their attitudes toward various learning modalities. Next, participants were fitted with an EEG headset. The device was placed such that the participant was comfortable and a signal connection was established on the Emotiv software (Emotiv Inc., San Francisco, CA). The participant then began their first randomized learning session while the EEG recorded brain activity. A maximum of 50 minutes was given for each learning session. After participants completed each of the sessions, the EEG recording for that session was terminated. Participants then completed a posttest - promptly after the learning session. The posttest evaluated immediate knowledge gain, their satisfaction with the teaching, and an assessment of the cognitive load while learning under each condition. This procedure of learning intervention followed by an immediate posttest was repeated for the two other learning interventions. A follow-up knowledge test was sent four weeks after the study day via email to assess knowledge retention. All data collection was completed between January and April 2021. Our medical school’s institutional review board approved this study.

Measures

Primary outcome variables included knowledge and attention under each condition. Participants’ immediate gains and long-term retention were measured by a knowledge test administered at three-time points. These tests consisted of 13-16 multiple-choice questions (a total of 43 items across all three tests) pulled from banks of prior American Board of Anesthesiology (ABA) in-training exams and pilot tested for difficulty and discrimination. Participants’ level of attention under each learning condition was measured by EEG indices of attentional network activations.

The secondary outcome variables were participants’ satisfaction with learning under each of the conditions, as measured by a satisfaction questionnaire post-intervention, and perception of cognitive load with each learning condition, as assessed by a self-report NASA Task Load Index.

EEG data acquisition and processing

The participants were fitted with the wireless EMOTIV EPOC X (Emotiv Inc., San Francisco, CA) headset with 14 channels. The channel names according to the 10-20 system are as follows: AF3, F7, F3, FC5, T7, P7, O1, O2, P8, T8, FC6, F4, F8, and AF4.

The channels are mastoid-referenced. The device sequentially samples the 14 channels. Data is filtered and the output is a 256 Hz clean signal in the 0.2-45 Hz range [10]. A number of studies showed that this EEG headset was effective in acquiring research-grade data for studying attention, auditory event-related potentials, neurofeedback games for enhancing attention and memory, and inter-brain synchrony in teams across different settings, learners, and tasks [11-14].

**Figure 1:**
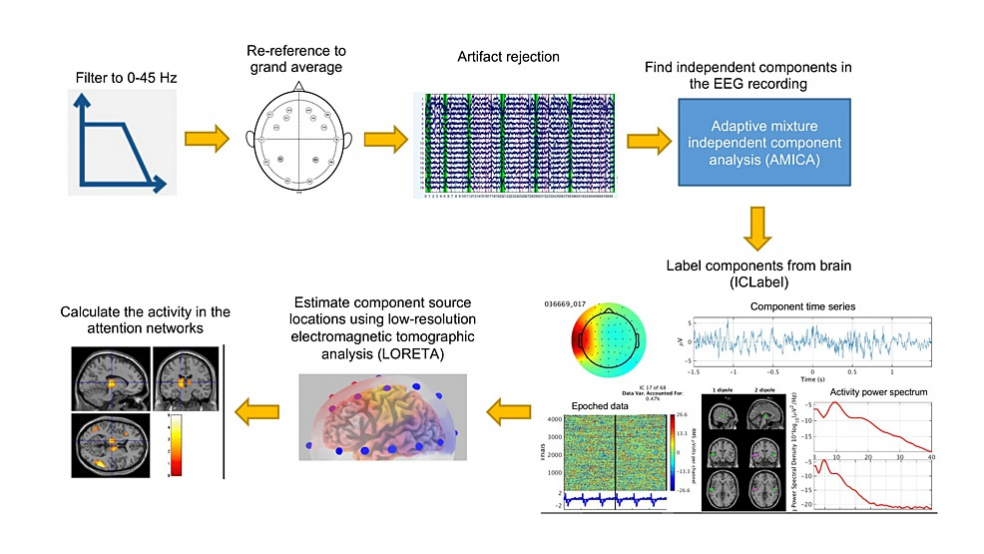
EEG data processing steps. First, EEGLAB is started and data is loaded to EEGLAB workspace. Any DC baseline is removed from the data, then data is bandpass filtered to 0.3-45 Hz range. Channels are re-referenced to grand average. Then, a mixed automatic/manual artifact rejection is employed. For the automatic rejection, contiguous data epochs are extracted and a standard spectrum thresholding algorithm is applied. Regions of contiguous epochs whose spectrum amplitude is larger than 10 dB in 0-10 and 35-45 Hz are then labeled as artifactual. Following this automatic procedure, data is manually inspected to accept/reject the results of the automatic artifact rejection. Then, independent component analysis (ICA) is performed. Next, the automatic EEG independent component classifier plugin for EEGLAB (https://labeling.ucsd.edu/tutorial/about) is use to label the components as brain and non-brain sources. Then, each component is mapped to source space using LORETA. LORETA models the cortex in 25892 voxels. This provides the opportunity to calculate the total activity level at the attention networks. LORETA: low-resolution electromagnetic tomographic analysis

This specific EEG headset uses Ag/AgCl electrodes, and the electrode was connected to the scalp via wet felts soaked in saline solution; impedance was measured to ensure the connection quality. The EEG data were recorded in the European data format (EDF) file for subsequent analysis. EEG data processing was carried out using EEGLAB [15-18]. The processing steps used are depicted in Figure 1.

Data analysis

We report descriptive questionnaire responses as frequencies and percentages. To allow for comparisons between knowledge scores for the three study topics, we converted scores for each topic to percentages by dividing each raw score by its maximum possible score. The immediate and long-term effects of learning conditions on each topic were examined by using a set of mixed-design ANOVAs with three data points (pre, post, follow-up) as within-subject factors, and three learning conditions as between-subject factors. Differences in EEG indices and cognitive load levels under the three distinct learning conditions were compared by using a set of repeated measures ANOVAs. We examined whether topics made a difference in the attentional network activation for each study condition separately. Since a set of one-way between-subject ANOVAs showed no significant effect on the topics for any of the conditions, we combined all topics for subsequent comparisons between study conditions using repeated measures ANOVAs. Simple main effects analyses or post-hoc tests with Bonferroni correction were used where applicable. Likert scale responses assessing satisfaction with each learning condition were analyzed with the non-parametric Kruskal-Wallis test. All statistical analyses were conducted with SPSS for Mac, version 27.0 (IBM Corp., Armonk, NY), with significance level set at p<0.05.

## Results

Of 65 participants, four were lost to follow-up, leaving a total of 61 (11 anesthesiology residents and 50 medical students) in the final analyses. Of the 61, six had corrupted EEG recordings and were excluded from the EEG analyses.

Some participants (17, 28%) reported that they almost never use podcasts for learning, but the majority (38, 62%) reported listening to podcasts at least one to two times a month. Most participants (42, 69%) also reported using podcasts while commuting, exercising, or doing chores around the house. Table 1 below shows the participant demographics.

**Table 1:** Participant characteristics and their use of podcasts for learning. "Unknown" (for age) indicates people who did not report their age. MS: medical school

Characteristic		N (%)
Sex	Male	33 (54)
	Female	28 (46)
Training level	MS years 1 and 2	19 (31)
	MS years 3 and 4	31 (51)
	Resident	11 (18)
Age (years)	25-29	29 (47)
	30-35	24 (39)
	36-40	4 (7)
	Unknown	4 (7)
Frequency of podcast use	Almost never	17 (28)
	1 to 2 times a year	6 (10)
	1 to 2 times a month	20 (32)
	1 to 2 times a week	12 (20)
	3+ times a week	6 (10)
How podcasts are used	I do not use podcasts	14 (23)
	While exercising/commuting/doing chores at home	42 (69)
	Just listen/watch podcast while doing nothing else	3 (5)
	Missing response	2 (3)

Knowledge scores

A set of three mixed ANOVA tests were conducted for each of the following included topics: allergic response, liver physiology, and statistics. For the mixed ANOVA tests, assumptions for Levene’s and Box’s M tests as well as Mauchly test of sphericity were met. We report these results for each of the topics below. 

Topic 1: allergic response

There was a significant interaction term for allergy scores between the three testing times and learning conditions as follows: F (4,116)=10.40, p<0.001, partial η^2^=0.26. This result indicates that the magnitude of scores differed significantly among the three learning conditions across pretest, posttest, and four-week follow-up.

When comparing the learning conditions, there were no differences in pretest scores. Participants listening to the podcast episode on allergic response while sitting and those listening to it when walking outperformed the participants in the book chapter learning condition at the immediate posttest (both p<0.001).

**Figure 2:**
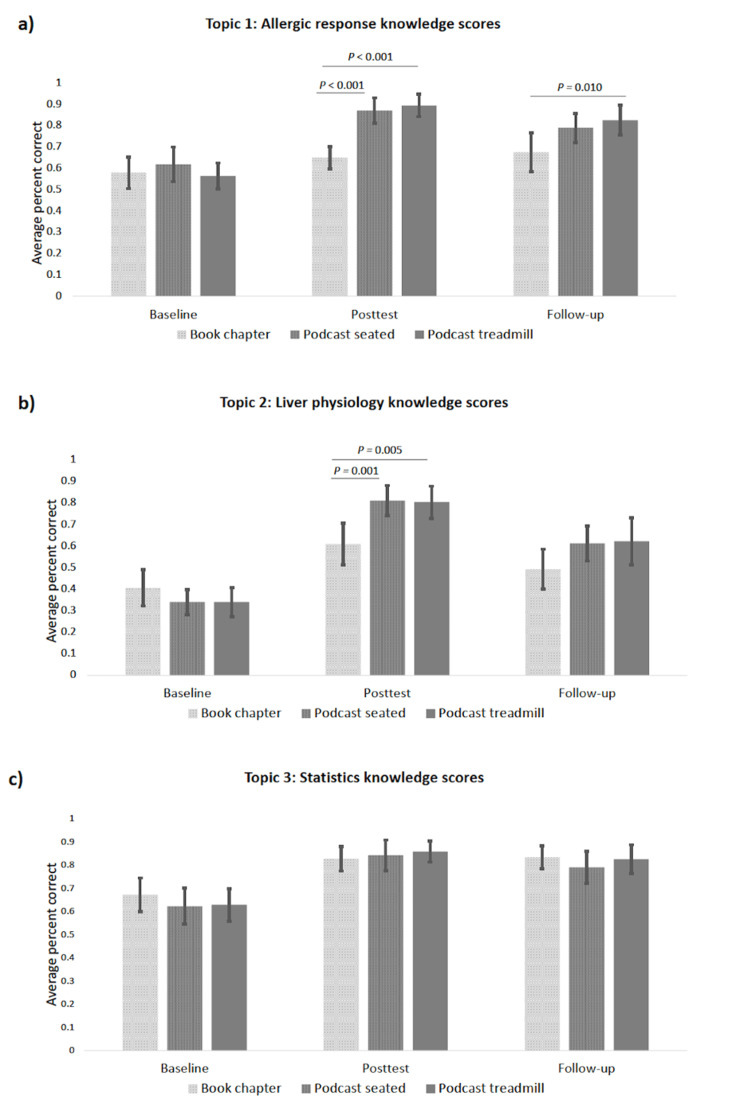
Average percent correct scores for (a) allergic response, (b) liver physiology, (c) statistics by learning conditions at pretest, posttest, and four-week follow-up.

Examining retention, participants in the podcast treadmill condition scored significantly higher than those reading a book chapter on allergic response at the four-week follow-up (p=0.01). There was a trend toward improved performance on the four-week follow-up test for podcast listening while seated versus reading a book chapter but it was not statistically significant (p=0.081) (Figure 2, panel a).

Topic 2: liver physiology

There was a significant interaction for liver knowledge test scores between the three testing times and learning conditions as follows: F (4,116)=10.01, p<0.001, partial η^2^=0.26. Simple main effects showed no differences between the learning conditions at pretest. On the immediate posttest, participants who listened to the podcast episode on liver physiology while seated or walking scored significantly higher than the participants who read the book chapter (p=0.001, p=0.005, respectively), but no difference was noted at four-week follow-up (p=0.132, p=0.199, respectively) (Figure 2, panel b).

Topic 3: statistics

Statistics knowledge test scores showed no significant interaction between the three testing times and learning conditions: F (4,114)=0.94, p<0.001, partial η^2^=0.03. There was a significant main effect for time (F {2,114}=80.09, p<0.001, partial η^2^=0.58). In all three conditions, participants had higher scores on the posttest than on the pretest (p<0.001) and maintained this increase at the four-week follow-up (p<0.001). There was no difference between any learning condition for the statistics topic (F {2,57}=0.28, p=0.755, partial η^2^=0.01) (Figure 2, panel c).

EEG measure of attention

**Figure 3:**
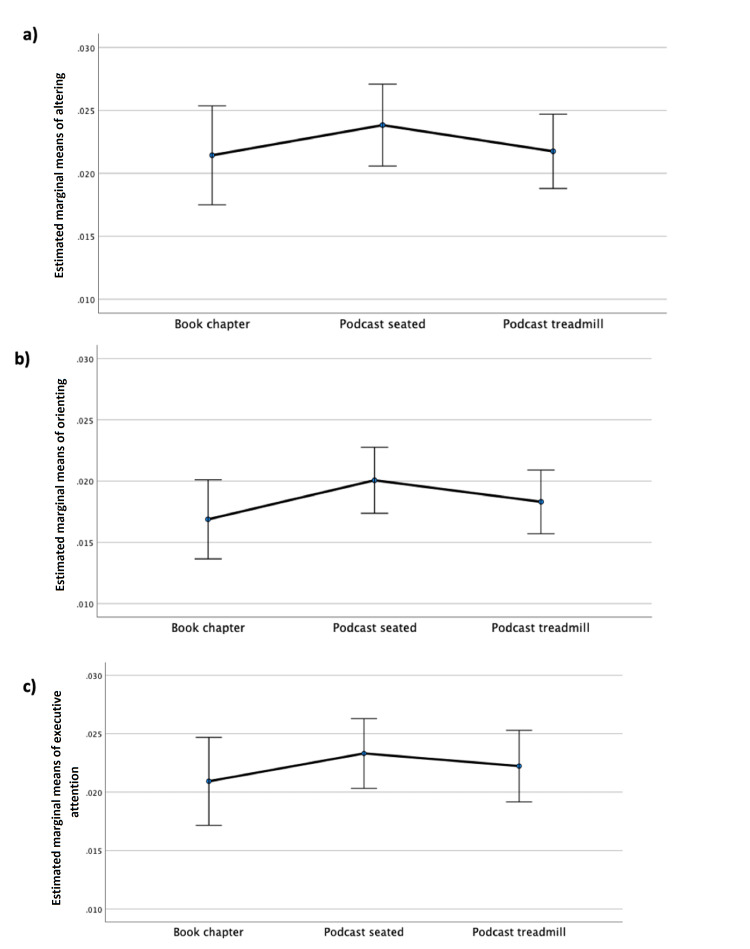
Comparisons between learning conditions for activation scores in each attentional network (a) alerting, (b) orienting, and (c) executive. There was no significant difference between any of the comparisons. Error bars indicate 95% confidence intervals.

For all repeated measures ANOVAs, the sphericity assumption was met, as Mauchly’s test was not significant. The mean attention network activation scores did not differ between the learning conditions for any of the attention networks as follows: alerting (F {2,108}=0.753, p=0.473) (Figure 3, panel a); orienting (F {2,108}=1.492, p=0.230) (Figure 3, panel b); and executive attention (F {2,108}=0.656, p=0.521) (Figure 3, panel c).

Participant satisfaction with learning condition

Participants rated reading a book chapter less favorable than podcast listening while seated for all of the content areas (all three p<0.05). They also found reading a book chapter less favorable than podcast listening on a treadmill (for two of the three topics {both p<0.001}), with a trend towards statistical significance for liver physiology learning (p=0.063). As for the self-reported task load, the participants reported higher perceived task load while reading a book chapter than while listening to a podcast seated or while walking for all content areas (all p<0.05).

## Discussion

In this cohort of volunteer learners at this institution, participants preferred listening to podcasts over reading a textbook. Beyond preference, podcast learning improved posttest knowledge acquisition in two out of three topics tested and showed a trend toward improved retention. Learners had equivalent EEG-measured attention with podcasts and textbooks, even when listening to a podcast while walking on a treadmill. These data may explain why podcasts are flourishing.

Our findings add to the existing literature suggesting that some learners may prefer podcasts over other forms of learning, such as textbooks, at least for some topics and settings [1,19-21]. Indeed, the popularity of podcasts has led many academic journals to launch podcasts of their own to attract broader audiences [22]. Vasilopoulos et al. found that anesthesia residents learning to read EEGs did better when given a podcast overview of the process than they did when given a traditional lecture [23]. Various other studies have similarly suggested a potential benefit in terms of learning outcomes as measured by exam performance when comparing podcasts to lectures or textbooks [24-27]. Our findings support these studies as our learners performed better on posttests after listening to podcasts than after reading a textbook for two of the three topics. While we cannot tell from our study, it is possible that no difference was found for the statistics topic because this topic is less conducive to audio-only learning. Further studies could examine whether certain topics, such as this one, are better taught through one modality or another.

Our EEG findings are unique in the anesthesiology and podcast education literature. Our findings contribute to the growing body of literature supporting the use of EEG data to evaluate different aspects of learning [6]. It is feasible to measure EEG waveforms even while participants are moving, in this case on a treadmill, suggesting that this modality can likely be used to evaluate attention during activities that require movements, such as during simulated clinical scenarios or even actual clinical work. While Riddell’s study suggested that some learners may be distracted by multitasking while listening to podcasts, our EEG data shows that, at least while walking, learners showed similar levels of attention as when reading a textbook [2]. This is not inconsistent with our performance data.

Grammer et al. in 2021 used EEG for comparing the effects of different educational modalities on student attention in an undergraduate education setting [28]. This study used lower alpha and higher beta and gamma spectral powers as markers for attention and found that student-led activities (group or individual work) were associated with stronger attention than teacher-led (lecture or video) activities. However, attention is a complex neurocognitive process involving multiple regions of the brain depending on the nature of the cognitive task. A study by Fan et al. in 2007 combined both functional magnetic resonance imaging (fMRI) and EEG data and found that it may be more effective to examine the distinct oscillatory activity within attentional networks rather than analyzing specific frequency bands [29]. In fact, based on decades of research, Posner and Fan in 2008 argue that attention can be seen as an organ system as three distinct brain regions can be consistently associated with following three different attentional functions: alerting, orienting, and executive [30]. Thus, we believe examining activation levels in these distinct attentional networks provides powerful quantitative measures of attention.

Several limitations of this study should be considered. First, while we opened enrollment to all medical students and anesthesiology residents, we closed the sampling pool once we reached the target n based on sample size calculations. It is possible that those who signed up may like podcasts, and thus be more likely to prefer them, than those who did not sign up. Given that individuals served as their own controls in the design, any differences from peers may be less relevant. Second, the study was conducted at a single institution; it is possible that residents and students at other institutions may have different preferences for learning. Third, when knowledge assessments are delayed over time to assess retention, it is possible that experiences or curricular elements during that interval may have covered similar material. To the best of our knowledge, our participants did not have any teaching that specifically addressed any of the content areas. Finally, listening to a podcast while walking on a treadmill approximates real-world activity more so than sitting in a quiet laboratory, however, it does not simulate the diversity of the real-world experience that trainees may engage in while listening to a podcast on their own time.

## Conclusions

In contemporary medical education, asynchronous teaching through podcasts appears to be useful for knowledge acquisition and they are well received; further, they maintain learners’ attention even while they are engaged in physical activity. The use of EEG data to measure attention during medical education may be a valuable method for exploring how well innovative educational modalities engage trainees. While further research is necessary and some questions remain unanswered, educators can feel more confident in recommending podcasts as supplements to their curricular offerings. Learners may be reassured to understand that podcasts can support knowledge acquisition that can be absorbed and retained.

## References

[1] Cadogan M, Thoma B, Chan TM, Lin M (2014). Free open access meducation (FOAM): the rise of emergency medicine and critical care blogs and podcasts (2002-2013). Emerg Med J.

[2] Riddell J, Robins L, Brown A, Sherbino J, Lin M, Ilgen JS (2020). Independent and interwoven: a qualitative exploration of residents' experiences with educational podcasts. Acad Med.

[3] Lee J, Lin L, Robertson T (2012). The impact of media multitasking on learning. Learn Media Technol.

[4] Andrade J (2010). What does doodling do?. Appl Cognit Psychol.

[5] Hötting K, Schickert N, Kaiser J, Röder B, Schmidt-Kassow M (2016). The effects of acute physical exercise on memory, peripheral BDNF, and cortisol in young adults. Neural Plast.

[6] Toy S, Huh DD, Materi J, Nanavati J, Schwengel DA (2022). Use of neuroimaging to measure neurocognitive engagement in health professions education: a scoping review. Med Educ Online.

[7] Fan J, McCandliss BD, Fossella J, Flombaum JI, Posner MI (2005). The activation of attentional networks. Neuroimage.

[8] Fan J, McCandliss BD, Sommer T, Raz A, Posner MI (2002). Testing the efficiency and independence of attentional networks. J Cogn Neurosci.

[9] Barash PG, Cullen BF, Stoelting RK (2017). Clinical Anesthesia. Eight Edition. Clinical Anesthesia, ed. Eighth.

[10] (2022). EPOC+ user manual. https://emotiv.gitbook.io/epoc-user-manual/.

[11] Alchalcabi AE, Eddin AN, Shirmohammadi S (2017). More attention, less deficit: wearable EEG-based serious game for focus improvement. 2017 IEEE 5th International Conference on Serious Games and Applications for Health (SeGAH).

[12] Badcock NA, Preece KA, de Wit B, Glenn K, Fieder N, Thie J, McArthur G (2015). Validation of the Emotiv EPOC EEG system for research quality auditory event-related potentials in children. PeerJ.

[13] Esqueda-Elizondo JJ, Juárez-Ramírez R, López-Bonilla OR (2022). Attention measurement of an autism spectrum disorder user using EEG signals: a case study. Math Comput Appl.

[14] Reinero DA, Dikker S, Van Bavel JJ (2021). Inter-brain synchrony in teams predicts collective performance. Soc Cogn Affect Neurosci.

[15] Delorme A, Makeig S (2004). EEGLAB: an open source toolbox for analysis of single-trial EEG dynamics including independent component analysis. J Neurosci Methods.

[16] Palmer Palmer, J J (2022). AMICA - adaptive mixture ICA. https://sccn.ucsd.edu/~jason/amica_web.html.

[17] Pion-Tonachini L, Kreutz-Delgado K, Makeig S (2019). ICLabel: an automated electroencephalographic independent component classifier, dataset, and website. Neuroimage.

[18] Pion-Tonachini L, Makeig S, Kreutz-Delgado K (2017). Crowd labeling latent Dirichlet allocation. Knowl Inf Syst.

[19] Mallin M, Schlein S, Doctor S, Stroud S, Dawson M, Fix M (2014). A survey of the current utilization of asynchronous education among emergency medicine residents in the United States. Acad Med.

[20] Riddell J, Swaminathan A, Lee M, Mohamed A, Rogers R, Rezaie SR (2017). A survey of emergency medicine residents' use of educational podcasts. West J Emerg Med.

[21] Wolpaw J, Toy S (2018). Creation and evaluation of an anesthesiology and critical care podcast. J Educ Perioper Med.

[22] Trueger NS (2018). Medical journals in the age of ubiquitous social media. J Am Coll Radiol.

[23] Vasilopoulos T, Chau DF, Bensalem-Owen M, Cibula JE, Fahy BG (2015). Prior podcast experience moderates improvement in electroencephalography evaluation after educational podcast module. Anesth Analg.

[24] Alam F, Boet S, Piquette D, Lai A, Perkes CP, LeBlanc VR (2016). E-learning optimization: the relative and combined effects of mental practice and modeling on enhanced podcast-based learning-a randomized controlled trial. Adv Health Sci Educ Theory Pract.

[25] Alla A, Kirkman MA (2014). PodMedPlus: an online podcast resource for junior doctors. Med Educ.

[26] Florescu CC, Mullen JA, Nguyen VM, Sanders BE, Vu PQ (2015). Evaluating didactic methods for training medical students in the use of bedside ultrasound for clinical practice at a faculty of medicine in Romania. J Ultrasound Med.

[27] Kurien G, Biron VL, Campbell C, Cote DW, Ansari K (2013). Can a multisensory teaching approach impart the necessary knowledge, skills, and confidence in final year medical students to manage epistaxis?. J Otolaryngol Head Neck Surg.

[28] Grammer JK, Xu K, Lenartowicz A (2021). Effects of context on the neural correlates of attention in a college classroom. NPJ Sci Learn.

[29] Fan J, Byrne J, Worden MS, Guise KG, McCandliss BD, Fossella J, Posner MI (2007). The relation of brain oscillations to attentional networks. J Neurosci.

[30] Posner MI, Fan J (2008). Attention as an organ system. Topics in Integrative Neuroscience.

